# Highly Pathogenic Swine Getah Virus in Blue Foxes, Eastern China, 2017

**DOI:** 10.3201/eid2506.181983

**Published:** 2019-06

**Authors:** Ning Shi, Li-Xia Li, Rong-Guang Lu, Xi-Jun Yan, Hao Liu

**Affiliations:** Foshan University, Foshan, China (N. Shi, H. Liu);; Jilin Wildlife Rescue and Rehabilitation Center, Forestry Department of Jilin Province, Changchun, China (L.-X. Li);; Chinese Academy of Agricultural Sciences, Changchun (R.-G. Lu, X.-J. Yan)

**Keywords:** Getah virus, blue fox, swine, China, phylogenetic analysis, viruses, mosquitoborne diseases, vector-borne infections, zoonoses

## Abstract

We isolated Getah virus from infected foxes in Shandong Province, eastern China. We sequenced the complete Getah virus genome, and phylogenetic analysis revealed a close relationship with a highly pathogenic swine epidemic strain in China. Epidemiologic investigation showed that pigs might play a pivotal role in disease transmission to foxes.

Getah virus (GETV; genus *Alphavirus*, family *Togaviridae*) is a mosquitoborne RNA virus that causes death in young piglets, miscarriage in pregnant sows, and mild illness in horses (*1**–**3*). Serologic surveys show that the infection might occur in cattle, ducks, and chickens (*4*); some evidence suggests that GETV can infect humans and cause mild fever (*5**,**6*).

In September 2017, twenty-five 5-month-old blue foxes at a farm in Shandong Province in eastern China showed symptoms of sudden fever, anorexia, and depression; 6 of the 25 animals had onset of neurologic symptoms and died on the third day of illness. We collected blood samples from 45 healthy and 25 ill foxes. We subjected the tissue samples from dead animals, including the brains, lungs, spleens, kidneys, livers, intestines, hearts, and stomachs, to hematoxylin and eosin staining. Microscopic examination confirmed the presence of typical lesions in cerebral cortices with mild neuronal degeneration and inflammatory cell infiltration in vessels, as well as severe hemorrhagic pneumonia, congestion, and hemorrhage with a large number of erythrocytes in the alveolar space (Figure) (*1*). No obvious lesions were found in other organs.

**Figure:**
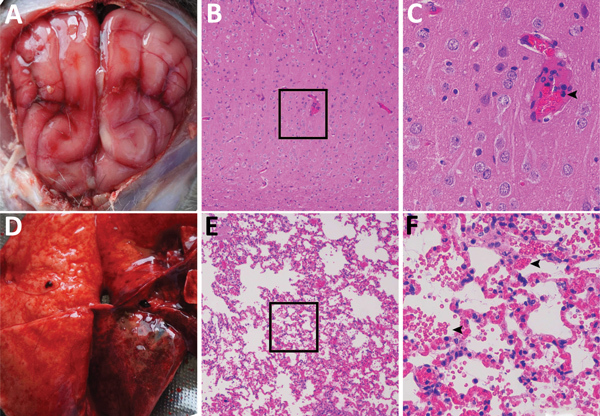
Dissected brain and lung of a dead fox, collected in 2017 in Shandong Province, eastern China, and histopathologic examination of samples using hematoxylin and eosin staining. A) Brain, showing congestion in the meninx. B) Histologic view of meninx, showing mild neuronal degeneration and inflammatory cell infiltration in vessels. Original magnification ×100. Box indicates area enlarged in panel C. C) A higher magnification view (original magnification ×400) of lesions in panel B, showing inflammatory cell infiltration in a vessel (arrow). D) Lung tissue, showing extensive congestion and hemorrhage. E) Histologic view of lung tissue, showing congestion, hemorrhage, or both, with many erythrocytes in the alveolar space. Original magnification ×100. Box indicates area enlarged in panel F. F) A higher magnification view (original magnification ×400) of tissue lesions in panel E, showing erythrocytes in the alveolar space (arrows).

We used supernatants of homogenized brain and lung tissues from each dead fox to inoculate Vero cells, as described previously (*7*). We observed a cytopathogenic effect within 72 hours. We observed numerous spherical, enveloped viral particles, ≈70 nm in diameter, after negative staining in a transmission electron microscope. To identify potential viral pathogens, we performed reverse transcription PCR (RT-PCR) to detect a panel of viruses, including canine distemper virus, canine parvovirus, canine coronavirus, and canine adenovirus. However, we detected none of these classical endemic viruses. 

During the investigation, farmers reported that the foxes had been fed on organs from symptomatic pigs. We therefore tested for the presence of African swine fever virus, pseudorabies virus, porcine reproductive and respiratory syndrome virus, classical swine fever virus, Japanese encephalitis virus, porcine circovirus type 2, porcine circovirus type 3, porcine cytomegalovirus, and alphavirus by using the primers for those viruses (Appendix Table 2). RT-PCR using universal primers for alphavirus (M2w-cMw3) produced a 434-bp amplicon when we tested all samples from dead foxes. Sanger sequencing of the amplicon and a BLAST search (https://blast.ncbi.nlm.nih.gov/Blast.cgi) identified the sequence as that of GETV. 

To further investigate the epidemic GETV infection, we performed quantitative RT-PCR by using RNA from all fox samples, as described elsewhere (*7*). Lung samples from all 6 dead foxes were positive, whereas only 2 samples from the remaining 19 ill foxes were also positive. None of the samples from healthy foxes were positive (Appendix Tables 1, 3). We measured serologic neutralizing antibodies by using a GETV isolate from a symptomatic fox, as previously described (*8**,**9*). Results showed no neutralizing antibody (<1:2) in healthy blue foxes (group 1) and variable levels of neutralizing antibodies (1:2 to 1:256) in ill foxes (groups 2–4) (Appendix Table 3). Samples from ill foxes with lower antibody titers had higher copies of RNA (groups 2–4). Spearman correlation analysis revealed a significant negative correlation between antibody titers and viral RNA copy numbers (r^2^ = 0.952; p<0.01).

We obtained the complete genome of the novel GETV SD1709 strain (GenBank accession no. MH106780) by using a conventional RT-PCR method (*10*). SD1709 genome sequence comparisons showed high identity with the porcine GETV strain (HuN1) at the nucleotide (99.6%) and deduced amino acid (99.7%–99.8%) sequences (Appendix Table 4). Furthermore, phylogenetic analysis of the complete genome and structural protein E2 gene indicated that the SD1709 strain was most similar to the recent epidemic HuN1 strain, which had caused large numbers of piglet deaths, stillbirths, and fetal mummies in southern China in 2017 (*1*) (Appendix Figures 1, 2).

We also detected GETV infection in pig serum samples and in mosquitoes (*Culex tritaeniorhynchus*, *Anopheles sinensis*, and *Armigeres subalbatus*) collected in the same region. The infection rate in pigs detected by quantitative RT-PCR was 20.0% (4/20) and by serum neutralization was 75.0% (15/20). The minimum infection rate in mosquitoes was ≈1.09%; *C. tritaeniorhynchus* mosquitoes had a higher minimum infection rate (2.31%) compared with other mosquito species (0–0.80%). These results suggest that pigs and *C. tritaeniorhynchus* mosquitoes might play a role in transmitting highly pathogenic GETV to captive foxes in this region (Appendix Tables 5, 6).

In China, the disease caused by GETV has only been reported in pigs in Hunan Province, although the virus has been detected in mosquitoes in >10 provinces (*1**,**4*). Our study provides evidence that GETV can cause lethal infection in blue foxes. Investigation of transmission routes for GETV in animals might help to prevent outbreaks of GETV disease in China.

Appendix Additional information regarding highly pathogenic swine Getah virus in blue foxes, eastern China, 2017.
