# Supplementary material for: Highly Pathogenic Swine Getah Virus in Blue Foxes, Eastern China, 2017
Source: Emerg Infect Dis. 2019 Jun;25(6):1252–4. doi: 10.3201/eid2506.181983 (PMC6537705; doi:10.3201/eid2506.181983)
Supplement: Appendix — Additional information regarding highly pathogenic swine Getah virus in blue foxes, eastern China, 2017. [file 18-1983-Techapp-s1.pdf]

Article DOI: https://doi.org/10.3201/eid2506.181983

Highly Pathogenic Swine Getah Virus in Blue Foxes, Eastern China, 2017

Appendix

Appendix Table 1. Analysis of a Getah virus–infected blue foxes with Neurologic symptoms and pneumonia, China, 2017*

Sample material	Histopathologic finding	Real-time PCR (cycle threshold)†
Brain	Mild neuronal degeneration and inflammatory cells, infiltrate in vessel	– (>35)
Lung	Severe congestion and hemorrhage developed in capillary of alveolar septa, many erythrocytes observed in alveolar space	+ (26–30)
Spleen	NSML	– (>35)
Kidney	NSML	– (>35)
Liver	NSML	– (>35)
Intestine	NSML	– (>35)
Heart	NSML	– (>35)
Stomach	NSML	– (>35)

*NSML, no major microscopic lesions; –, negative; +, positive
†Negative result >35; positive result <35.

Appendix Table 2. Primers used in the present study

Virus	Primer name	Anneal site	Sequence(5′–3′)	Length of amplification	References*
CDV	F4854	Fsp	TCCAGGACATAGCAAGCCAACA	681 bp	(*1*)
	R5535		GGTTGATTGGTTCGAGGACTGAA		
CPV	555for	VP2	CAGGAAGATATCCAGAAGGA	583 bp	(*2*)
	555rev		GGTGCTAGTTGATATGTAATAAACA		
CCoV	CCoV-F	Hel	ACATGGTATATCTATGTGCGCAA	252 bp	(*3*)
	CCoV-R		TGCAAGGCGCACTTGAGAT		
CAV	CAV-F	E1A	TGTGCCCATCGACAAGGAA	433 bp	(*3*)
	CAV-R		CTAATAGAAGCGGCCCAACTG		
ASFV	CD2–2F	EP402R	TCTGTTGATTCCCCAACTATTACA	816 bp	(*4*)
	CD2–2R		ATGGCGGGATATTGGGTAGT		
PRV	PRV-F	gD	ATGCGGCCCTTTCTG	217 bp	(*5*)
	PRV-R		CGGTTCTCCCGGTATTTAAGC		
PRRSV	ORF5-F	ORF5	GGCGACCGTTTTAGCCTGTCTT	735 bp	(*6*)
	ORF5-R		ATCATTATTGGCGTGTAGGTG		
CSFV	CSFV1	E2	GCTCCTGGTTGGTAACCTCGG	508 bp	(*7*)
	CSFV2		TGATGCTGTCACACAGGTGAA		
JEV	JEV1	E	TGTGGACTTTTCGGGAAGGG	1015 bp	(*7*)
	JEV2		GGTGAACGGCTCTTCCTATG		
PCV2	F-PCV	ORF1	GCTGCCACATCGAGAAAG	565 bp	(*8*)
	R-PCV		GACAGCAGTTGAGGAGTACC		
PCV3	PCV3-F	*Cap*	TCCAAACTTCTTTCGTGCCGTAG	264 bp	(*9*)
	PCV3-R		GGCTCCAAGACGACCCTTATGC		
PCMV	PCMV-F	gB	CCCTGATCTTAAATGACGAGGACGTGAC	413 bp	(*8*)
	PCMV-R		ACCGTCTGAGAGAGACTGAACTTCTCTGACAC		
Alphavirus	M2w		YAGAGCDTTTTCGCAYSTRGCHW	434 bp	(*10*)
	cMw3	NS1	ACATRAANKGNGTNGTRTCRAANCCDAYCC		
	M2W2		TGYCCNVTGMDNWSYVCNGARGAYCC		

*Primer sequences used to amplify the several important virus infected Canidae and pigs in previous reports.

Appendix Table 3. RT-qPCR and Serum neutralization (SN) tests results of GETV in serum samples of bule foxes from Shandong, eastern China*

Groups†	Clinical symptoms	SN test results (no. samples)‡	RT-qPCR (copies/μL)‡
1	No symptoms	<1:2 (n = 45)	Negative
2	Fever, depression, anorexia, systemic neurolologic symptoms, dyspnea, and emesis; weak in appearance; ultimately died	1:2 (n = 1)	1.698 $\times 10^{3}$
		1:2 (n = 1)	1.445 $\times 10^{3}$
		1:2 (n = 1)	1.718 $\times 10^{3}$
		1:4 (n = 1)	4.266 $\times 10^{2}$
		1:8 (n = 1)	1.466 $\times 10^{2}$
		1:16 (n = 1)	1.432 $\times 10^{2}$
3	Fever, depression, anorexia	1:16 (n = 1)	4.764 $\times 10^{1}$
		1:16 (n = 1)	6.531 ×10°
		1:32 (n = 1)	Negative
		1:32 (n = 1)	Negative
		1:32 (n = 1)	Negative
		1:32 (n = 1)	Negative
		1:64 (n = 1)	Negative
4	Spontaneous clearance	1:64 (n = 6)	Negative
		1:128 (n = 5)	Negative
		1:256 (n = 1)	Negative

*RT-qPCR, quantitative reverse transcription polymerase chain reaction; SN, serum neutralization
†The collected samples were divided into Group 1 (<1:2), Group 2 (1:2–1:16), Group 3 (1:16–1:64), Group 4 (>1:64) and according to the neutralizing antibody titer>1:4 was positive.
‡Spearman correlation analysis showed significant negative correlation between the antibody titers and viral RNA copy numbers (r^2 = 0.952, p<0.01).

Appendix Table 4. Nucleotide and amino acid sequence identity (%) for the complete genomes between the isolates SD1709 from fox in this study and others

	SD1709, %				
		Nonstructural polyprotein		Structural polyprotein	
Virus isolates	Complete genome (nt)	nt	aa	nt	aa
12IH26	97.7	97.7	99.4	97.7	99.4
14-I-605-C1	97.7	97.6	99.3	97.7	99.4
14-I-605-C2	97.7	97.6	99.3	97.7	99.4
15-I-1105	97.6	97.6	99.1	97.6	99.3
15-I-752	97.7	97.6	99.2	97.7	99.4
16-I-599	97.7	97.6	99.2	97.6	99.3
16-I-674	97.6	97.6	99.1	97.6	99.3
16-I-676	97.6	97.6	99.1	97.6	99.3
GETV-V1	97.8	97.8	99.3	97.6	99.2
HB0234	97.8	97.8	99.1	97.6	99.0
HuN1	99.6	99.5	99.7	99.7	99.8
Kochi/01/2005	99.4	99.4	99.7	99.3	99.5
LEIV 16275 Mag	97.5	97.5	99.4	97.3	99.0
LEIV 17741 MPR	98.5	98.4	99.4	98.5	99.4
M1	97.9	98.0	99.1	97.7	98.3
MI-110-C1	98.5	98.4	99.6	98.5	99.4
MI-110-C2	98.5	98.4	99.6	98.5	99.4
ROK	98.1	98.1	99.6	98.1	99.4
Sagiyama virus	97.2	97.4	99.2	96.8	98.2
SC1210	97.5	97.8	99.4	97.6	99.2
YN0540	97.6	97.9	99.4	97.8	99.4
YN12031	96.3	96.3	98.8	96.1	98.2

Appendix Table 5. RT-qPCR and SN were used to detect pigs serum positive rates of GETV on different age group*

	RT-qPCR			SN		
Sampling age group	No. swine	No. swine testing positive	No. (%) of swine testing positive (95%CI)	No. swine	No. swine testing positive	No. (%) of swine testing positive (95%CI)
Nursery pigs	8	2	25.0 (23–26)	8	5	62.5 (41–83)
Fattening pigs	5	1	20.0 (18–21)	5	3	60.0 (15–104)
Sow	7	1	14.3 (13–15)	7	7	100.0 (54–145)
Total	20	4	20.0 (19–21)	20	15	75.0 (48–101)

*RT-qPCR, quantitative reverse transcription polymerase chain reaction; SN, serum neutralization.

Appendix Table 6. GETV infection in mosquitoes collected from Linyi of Shandong province, eastern China by RT-qPCR*

Species	No. mosquitoes	No. pools (100 mosquitoes/pool)	MIR of mosquitoes, % (no. positive pools/total specimens)†
Culex tritaeniorhynchus	1,300	13	2.31 (3/1300)
Anopheles sinensis	2,500	25	0.80 (2/2500)
Armigeres subalbatus	800	8	0.00 (0/800)
Total	4,600	46	1.09 (5/4600)

*RT-qPCR, quantitative reverse transcription polymerase chain reaction; MIR, minimum infection rate.
†MIR uses the assumption that a positive pool contains only 1 infected mosquito the minimum infection rate, which is calculated: ([number of positive pools/total specimens tested] x 1,000) (https://www.cdc.gov/westnile/resourcepages/mosqSurvSoft.html).

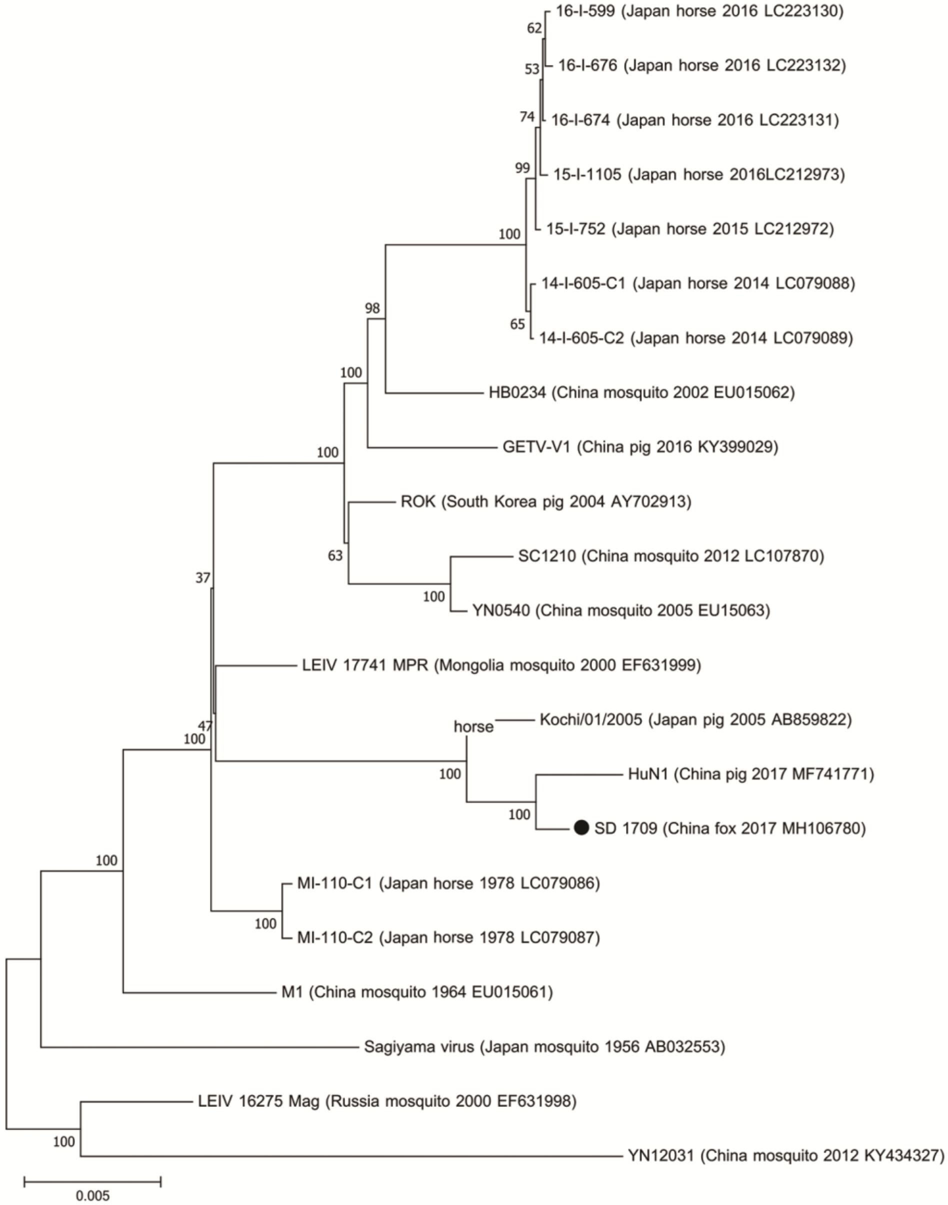

Appendix Figure 1. Phylogenetic analyses of the nucleotide sequences of the complete genome of Getah virus isolated in Shandong. Evolutionary history was inferred using the maximum likelihood method with the Tamura–Nei model and gamma-distributed rate heterogeneity in MEGA 7. The percentage of replicates in which the associated virus clustered together in the bootstrap test (1,000 replicates) is shown next to the branch in each tree. The strain isolated in this study is identified by ●. The percentage bootstrap support is indicated by the value at each node. Scale bar denotes nucleotide substitutions per site.

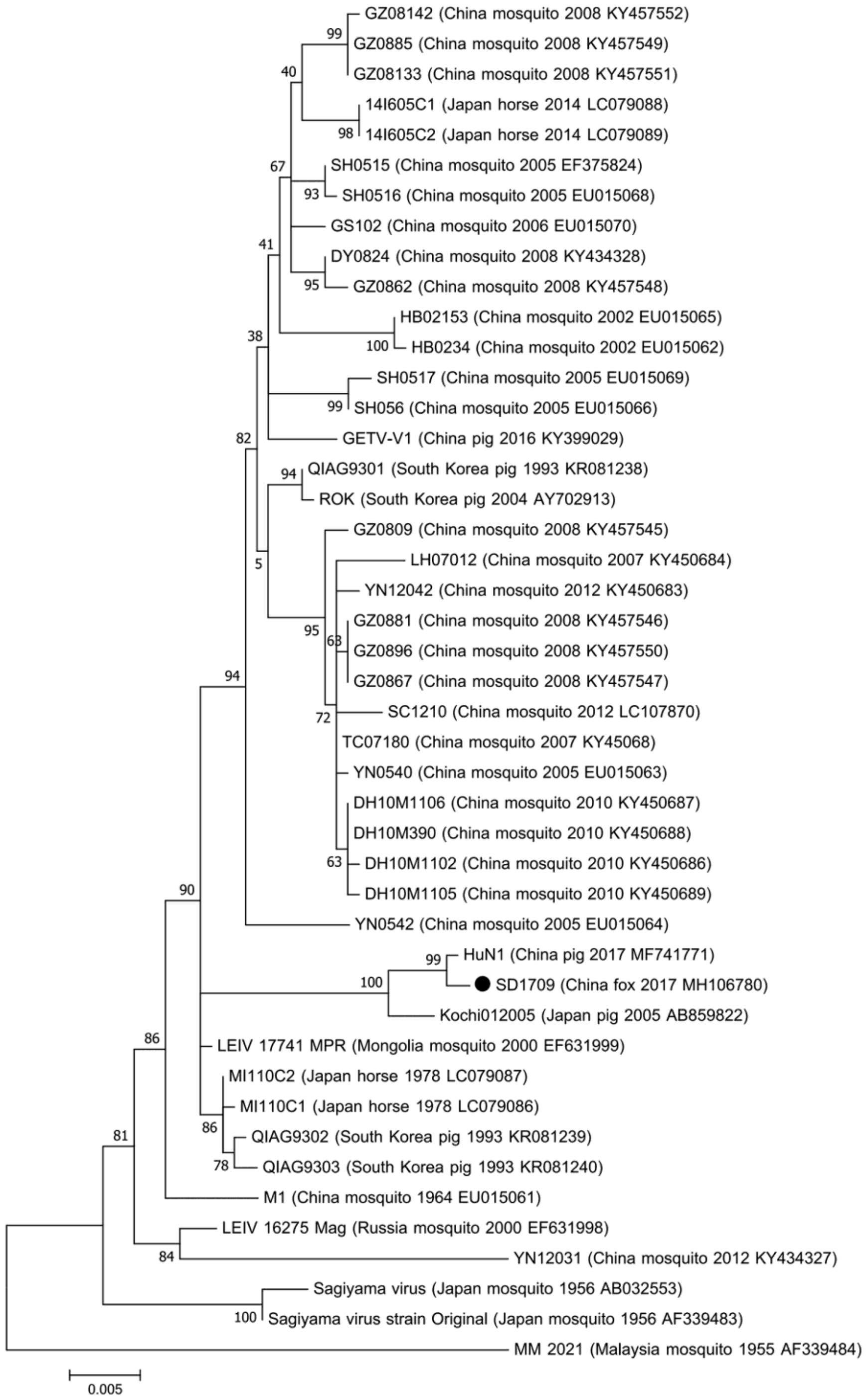

Appendix Figure 2. Phylogenetic analyses of E2 gene nucleotide sequences of Getah virus isolated in Shandong, 2017.The strain isolated in this study is identified by ●.

References

1. Sarute N, Calderón MG, Pérez R, La Torre J, Hernández M, Francia L, et al. The fusion protein signal-peptide-coding region of canine distemper virus: a useful tool for phylogenetic reconstruction and lineage identification. PLoS One. 2013;8:e63595. PubMed http://dx.doi.org/10.1371/journal.pone.0063595

2. Kapiya J, Nalubamba KS, Kaimoyo E, Changula K, Chidumayo N, Saasa N, et al. First genetic detection and characterization of canine parvovirus from diarrheic dogs in Zambia. Arch Virol. 2019;164:303–7. PubMed http://dx.doi.org/10.1007/s00705-018-4068-3

3. Deng X, Zhang J, Su J, Liu H, Cong Y, Zhang L, et al. A multiplex PCR method for the simultaneous detection of three viruses associated with canine viral enteric infections. Arch Virol. 2018;163:2133–8. PubMed http://dx.doi.org/10.1007/s00705-018-3828-4

4. Sanna G, Dei Giudici S, Bacciu D, Angioi PP, Giammarioli M, De Mia GM, et al. Improved strategy for molecular characterization of African swine fever viruses from Sardinia, based on analysis of p30, CD2V and I73R/I329L variable regions. Transbound Emerg Dis. 2017;64:1280–6. PubMed http://dx.doi.org/10.1111/tbed.12504

5. Liu H, Li XT, Hu B, Deng XY, Zhang L, Lian SZ, et al. Outbreak of severe pseudorabies virus infection in pig-offal-fed farmed mink in Liaoning Province, China. Arch Virol. 2017;162:863–6. PubMed http://dx.doi.org/10.1007/s00705-016-3170-7

6. Liang W, Zhao T, Peng Z, Sun Y, Stratton CW, Zhou D, et al. Epidemiological and genetic characteristics of porcine reproductive and respiratory syndrome virus circulating in central and South China in 2016. Acta Trop. 2019;190:83–91. PubMed http://dx.doi.org/10.1016/j.actatropica.2018.11.004

7. Zeng Z, Liu Z, Wang W, Tang D, Liang H, Liu Z. Establishment and application of a multiplex PCR for rapid and simultaneous detection of six viruses in swine. J Virol Methods. 2014;208:102–6. PubMed http://dx.doi.org/10.1016/j.jviromet.2014.08.001

8. Lee CS, Moon HJ, Yang JS, Park SJ, Song DS, Kang BK, et al. Multiplex PCR for the simultaneous detection of pseudorabies virus, porcine cytomegalovirus, and porcine circovirus in pigs. J Virol Methods. 2007;139:39–43. PubMed http://dx.doi.org/10.1016/j.jviromet.2006.09.003

9. Wen S, Sun W, Li Z, Zhuang X, Zhao G, Xie C, et al. The detection of porcine circovirus 3 in Guangxi, China. Transbound Emerg Dis. 2018;65:27–31. PubMed http://dx.doi.org/10.1111/tbed.12754

10. Pfeffer M, Proebster B, Kinney RM, Kaaden OR. Genus-specific detection of alphaviruses by a semi-nested reverse transcription-polymerase chain reaction. Am J Trop Med Hyg. 1997;57:709–18. PubMed http://dx.doi.org/10.4269/ajtmh.1997.57.709
